# Evaluation of the Efficacy of Play Therapy among Children Undergoing Dental Procedure through Drawings Assessed by Graphological Method: A Clinical Study

**DOI:** 10.5005/jp-journals-10005-1549

**Published:** 2018-10-01

**Authors:** Shital DP Kiran, Aayushi Vithalani, Devdatt J Sharma, Megha C Patel, Rohan Bhatt, Mohit Srivastava

**Affiliations:** 1 Professor and Head, Department of Pedodontics and Preventive Dentistry, Karnavathi School of Dentistry, Gujarat, India; 2 Postgraduate Student, Department of Pedodontics and Preventive Dentistry, Karnavathi School of Dentistry, Gujarat, India; 3 Postgraduate Student, Department of Pedodontics and Preventive Dentistry, Karnavathi School of Dentistry, Gujarat, India; 4 Reader, Department of Pedodontics and Preventive Dentistry, Karnavathi School of Dentistry, Gujarat, India; 5 Reader, Department of Pedodontics and Preventive Dentistry, Karnavathi School of Dentistry, Gujarat, India; 6 Senior Lecturer, Department of Pedodontics and Preventive Dentistry, Karnavathi School of Dentistry, Gujarat, India

**Keywords:** Anxiety, Behavioral management, Bubble play therapy

## Abstract

**Introduction:**

Behavior modification is defined as the attempt to alter human behavior and emotion in a beneficial way and accordance with the laws of learning. Play therapy is one such behavior modification technique. The study aimed to evaluate the efficacy of play therapy among children undergoing dental treatment by the graphological method.

**Materials and methods:**

Children were made to draw before treatment, after treatment of one class 1 lesion without application of any behavior modification technique and after treatment of the second lesion of class 1 caries with play therapy. Graphologist and scores assessed drawings were given by graphological method.

**Results:**

Significant reductions in stress levels were observed in the drawings which were made after play therapy.

**Conclusion:**

Play therapy is an effective behavior modification technique in pediatric dentistry, which may be used in routine dental practice.

**How to cite this article:** Kiran SDP, Vithalani A, Sharma DJ. Patel MC, Bhatt R, Srivastava M. Evaluation of the Efficacy of Play Therapy among Children Undergoing Dental Procedure through Drawings Assessed by Graphological Method: A Clinical Study. Int J Clin Pediatr Dent.,2018;11(5):412-416.

## INTRODUCTION

Anxiety is defined as the fear of unknown Dentistry involves many procedures that involve pain and anxiety as children are unaware of the new things that are going to happen to them. Anxiety may also prevail because of knowledge about past experiences of dental treatment of their family members. Maternal influence furthermore plays an important role.^1^ This pain, anxiety, fear, phobia, distress makes the child negative for the dental treatment and has the psychological effect on them. It becomes a major barrier to carry out further dental pro-cedures.^2^ The willingness of children is reduced for the dental treatment. There are various methods to evaluate the behavior of the children and their attitude towards dental treatment. Various anxiety measurement scales are also there, which predicts the need for the behavior modification.

Behavior modification is defined as the attempt to alter human behavior and emotion in a beneficial way and following the laws of learning.^3^ Behavior modification is utmost necessary for these types of children who had become negative for the dental treatment because of fear, anxiety, and distress. Various behavior modification techniques are there, which can be implied on different children. One of them is play therapy.

Play therapy can be defined as an interpersonal process wherein a trained therapist systematically applies the curative powers of play (e.g., relationship enhancement, role-playing, abreaction, communication, mastery, catharsis, attachment formation, etc.) to help the clients resolve their current psychological difficulties and help prevent future ones. Play therapy techniques specify how to use the play materials to implement the therapeutic powers of play effectively. Three main criteria guided the selection of techniques for this article: (a) to include an extensive variety of play approaches (e.g., sensorimotor, art, fantasy, and gameplay), (b) to focus on techniques appropriate for 4 to 12-year-old children, and (c) to present techniques that are enjoyable, inexpensive, and easy to implement. The goals of the chosen techniques include helping children become aware of express their feelings; manage anger; improve self-control; reduce fear, anxiety, and depression; increase empowerment; and enhance problem-solving skills.^4^

Procedural pain, anxiety, and distress is expressed and judged in many ways like facial expressions, child’s behavior, speech, body language, gait, their way of holding things, handwriting, drawings, their way of responding, etc. One such study is graphology, which deals with the prediction of a person’s psychology with the help of handwriting, signature, and drawings. Most of the children have always welcomed drawing and coloring activity. Post dental treatment drawings may be helpful for children in distraction from the pain and distress they have felt during the procedure.^5^

Assessment of anxiety levels and distress amongst children undergoing dental treatment would be done with the help of graphologically method from pre and post-treatment drawings made by children. This graphology will aid as a tool to measure the level of anxiety and distress. Thus the usefulness of the play therapy will also be judged by the variations seen at the anxiety level before and after instillating the therapy.

The activity of drawing is a subject of interest. Nevertheless, by most of the children, this activity is warmly welcomed. Drawing is a pleasant exercise that reflects their inner world, thought processes, feelings and emotions and psychological status, particularly at a certain point of time. Free drawings (child is free to draw anything without directions or instructions), bridge drawings (child is asked to draw about future expectations and relative threat), volcano drawings (child is asked to draw his/her means to manage anxiety), person picking an apple from a tree (to know the child’s coping ability and resourcefulness), kinetic family drawings (child asked about family dynamics), human figure drawings (asked to draw a picture of a person) are the various means employed in studies on children drawings, of which, human figure drawings are popular clinically.

To our best of knowledge, no study has been carried out regarding the assessment of distress in children undergoing dental procedures using the graphology.

## MATERIALS AND METHODS

The proposed study was conducted as the clinical psychology of behavior management study. Prior written permission and consent were obtained from the participant’s parents and also by theinstitutional ethical committee.

### Sample

All the samples were selected from the outpatient department (OPD) of the department of pedodontics and preventive dentistry. The study was conducted between 25 patients achieved by 95% power with a known standard deviation of 1.25, and the level of significance was set at 0.05. The children aged between 5-10 years whose parents gave the consent and who met all the inclusion criteria.

### Inclusion Criteria

- Children aged between 5 to 10 years.
- Children having class one dental caries in at least two teeth.
- Physically and mentally fit children.
- Children without any developmental or congenital abnormalities.
- Children whose parents will give written consent for the study.

### Exclusion Criteria

- Children with physical or psychological abnormalities.
- Children with developmental or congenital abnormality.
- Children below age from 5 years or above 10 years.
- Children whose parents refuse to give the written consent.
- Other than children having class one dental caries in at least two teeth.
- Children not willing to make drawings.
- Class 1 dental caries which will have to be treated with indirect pulp capping.

### Methodology

After considering all the inclusion and exclusion criteria, a total of 25 participants (boys and girls both) were selected into the study.

All the participants were roughly examined by the principal investigator and the children who meet the requirement. They were be given A4 size drawing sheets, pencil, and stationery box and they were made to draw any random pictures of their choice without anyone’s supervision. After that, out of two carious lesions, and one lesion of class one dental caries was treated without applying any behavior modification technique. In the treatment of one lesion, caries excavation was done, and composite or glass ionomer cement restoration was given. Then the child wastaken to the play area after treatment of one carious lesion and was instructed to make the similar kind of drawing that he made previously again without anyone’s supervision. Next, the child’s behavior was modified with play therapy behavior modification technique using bubble breath and 2nd lesion of class one dental caries was treated with composite or glass ionomer cement restoration. Lastly, the child was made to draw the picture again with the same instructions. In the treatment of both the teeth, first caries excavation was done, and the cavity was reviewed. If it was too deep and there was needed for indirect pulp capping, then those children were excluded from the study. These exclusion criteria were applicable to both of the teeth, during treatment.

These three drawings that is one pre-treatment and two post-treatment drawing was assessed by the graphologist having experience of 8 years. From these drawing’s assessment of anxiety level was done before treatment, after treatment without any behavior modification technique and post-treatment with play therapy. Thus, assessment of the efficiency of play therapy behavior modification technique was made by comparing these three pre and post-treatment drawings.

## STATISTICAL ANALYSIS

The collected data were entered and analyzed with SPSS 18.0 version. Descriptive statistical tests and inferential statistics were computed using excel statistical operations. Wilcoxon signed-rank test and paired sample t-test at 5% level of significance were set.

## RESULTS

**Graph 1:**
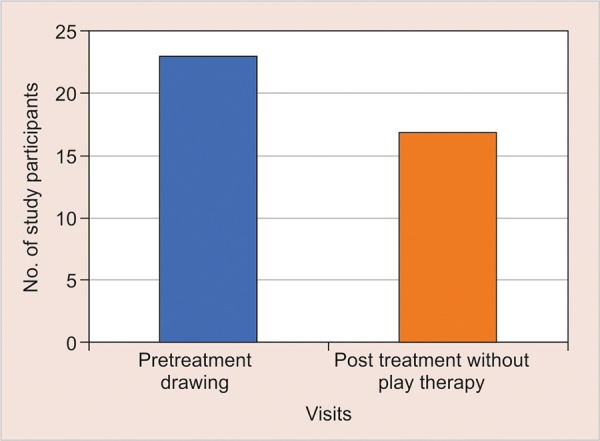
Comparison of anxiety level from scores of pre treatment drawing and post treatment drawing without play therapy

The mean anxiety level of children from pre-treatment drawing was 22.92 and post-treatment drawings without play therapy were 16.84 which indicatean increase in anxiety level. Thus there was a significant mean difference of -6.08 with the p < 0.001 (Table 1 and Graph 1).

**Graph 2:**
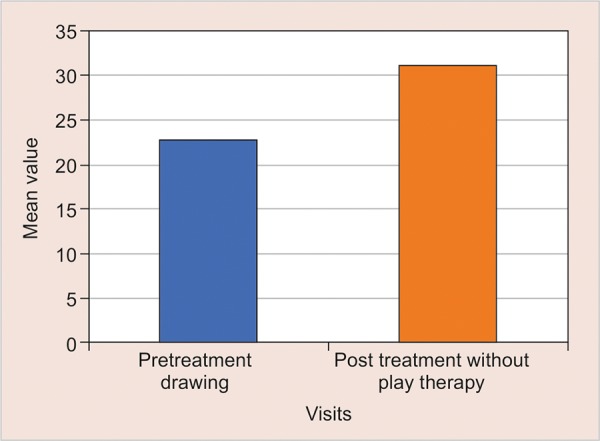
Comparison of anxiety level from scores of pre treatment drawing and post treatment drawing with play therapy

From the pretreatment drawing, mean anxiety level of children was 22.92 and post-treatment drawings with play therapy is 31.32. The difference in the mean anxiety level shows there is a decrease in anxiety level compared to baseline. Thus there was a significant mean difference of 8.40 with the level of significance < 0.001 (Table 2 and Graph 2).

**Graph 3:**
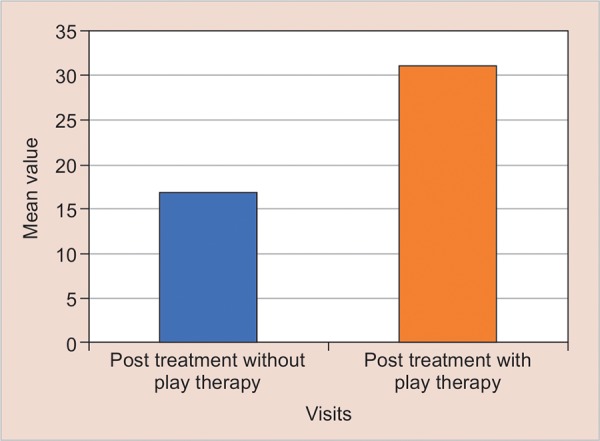
Comparison of anxiety level from scores of post treatment drawing without play therapy and post treatment drawing with play therapy

The mean anxiety level of children from post-treatment drawing without play therapy was 16.84 and post-treatment drawings with play therapy is 31.32. This indicates there is a decrease in anxiety level. Thus there was a significant mean difference of 14.48 with the level of significance <0.001 (Table 3 and Graph 3).

**Graph 4:**
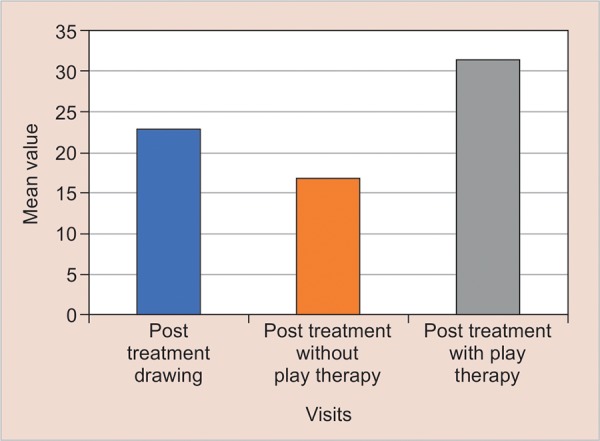
Comparison of anxiety level from scores of pre treatment drawing, post treatment drawing without play therapy and post treatment drawing with play therapy

It was observed that anxiety had increased in 2nd drawing and later it had decreased in 3rd drawing upto the extent that there was a significant difference when compared to baseline (Graph 4).

## DISCUSSION

Nonpharmacological behavior management techniques play a vital role in pediatric dentistry. Reframing is defined as, “taking a situation outside the frame that up to that moment contained the individual in different conditions and visualize (reframe) it in a way acceptable to the person involved and with this reframing, both the original threat and the threatened “solution” can be safely abandoned.”^6^

There are various techniques of play therapy namely spy and sneak, weights and balloons, bubble breath, color your life, the pickup sticks game, etc.^4^ Bubble breath play therapy offers more acceptance as children are familiar with the game and also provides relaxation technique for control of anxiety.

Among dental procedures, treatment of class I lesion was selected because it is a noninvasive and children can be controlled when the first lesion was treated without application of any other type of behavior modification technique. Children under 5 years of age would not be tackled when treatment was to be carried out without behavior modification. Moreover, children over 10 years of age might not show interest in bubble breath and efficacy of play therapy cannot be assessed without their involvement. Also, the acceptable age group for play therapy is 3 to 16,^7^ and 4 to 12 years.^4^ Therefore, in our study, children more than 5 years and less than 10 years were selected.

**Table 1:** **Table 1:** Comparison of anxiety level from scores of pretreatments drawing and posttreatment drawing without play therapy

		*Mean*		*Std. deviation*		*Std. error mean*		*Mean difference*		*p-value*	
Pretreatment drawing		22.92		3.45		0.690		–6.08		<0.001	
Posttreatment without play therapy		16.84		3.88		0.776					

**Table 2:** **Table 2:** Comparison of anxiety level from scores of pre treatment drawing and post treatment drawing with play therapy

		*Mean*		*Std. deviation*		*Std. error mean*		*Mean difference*		*p-value*	
Pre treatment drawing		22.92		3.45		0.690		8.40		<0.001	
Post treatment without play therapy		31.32		4.90		0.979					

**Table 3:** **Table 3:** Comparison of anxiety level from scores of post treatment drawing without play therapy and post treatment drawing with play therapy

		*Mean*		*Std. deviation*		*Std. error mean*		*Mean difference*		*p-value*	
Pretreatment drawing		16.84		3.88		0.776		14.48		<0.001	
Posttreatment without play therapy		31.32		4.90		0.979					

Morphological analysis of handwriting and handwritten free associations (couplings) as to the teeth may be a useful tool in the understanding of deeper psychological processes behind the phenomena of dental fear and anxiety.^8^

Different parameters of drawings predict anxiety levels.^9,10^ For e.g., red color depicts anger, brown and orange depicts fear whereas green color indicates calm state and blue indicates the more creative state of mind. Increased pressure and less clarity in drawing and coloring activity indicates more stress.More repetition of objects or colors and breaking lines depicts boredom.

In 1st drawing children were instructed to make any random pictures so that their anxiety level can be assessed, which prevails in them because of their surrounding environment. A patient coming to the dental clinic for the first time will be having full of mixed emotions, which were shown in (baseline drawing) 1st drawing where the anxiety level was moderate (mean value = 22.92). After treatment of 1 lesion of class I caries without application of any behavior modification technique, anxiety level increases to a significant level (mean value = 16.84) (p < 0.001). After the bubble breath play therapy, treatment was carried out, and thus the anxiety level decreased to a significant level (mean value = 31.32) with p-value p < 0.001. Play therapy demonstrated to be effective in reducing the anxiety which prevailed at the beginning also, i.e. pretreatment drawing and post-treatment after play therapy drawings showed significantly decreased of anxiety level (p < 0.001). This significant increase in anxiety level in the 2nd drawing is due to a treatment being done without application of any behavior modification technique whereas bubble breath play therapy was proved to be so significant that it had also reduced the baseline anxiety.

There are many advantages to drawing as it does not require any special training, not time consuming, easily accessible and inexpensive.^11^ Children after the painful activity got their attention for the drawing and left the dental clinic with an elevated mood.

## CONCLUSION

Bubble breath play therapy was found to be highly significant in reducing anxiety and distress among children undergoing the routine dental procedure. Play therapy is a simple, inexpensive, noninvasive, which can be used to modify the behavior in everyday pediatric dental practice. Inner emotional disturbances following the traumatic dental treatment has been proved in this study, and also this helps to shape up behavior management technique in the subsequent dental visit.
